# QueDI: From Knowledge Graph Querying to Data Visualization

**DOI:** 10.1007/978-3-030-59833-4_5

**Published:** 2020-10-27

**Authors:** Renato De Donato, Martina Garofalo, Delfina Malandrino, Maria Angela Pellegrino, Andrea Petta, Vittorio Scarano

**Affiliations:** 8 Linköping University, Linköping, Sweden; 9 University of Amsterdam, Amsterdam, Noord-Holland The Netherlands; 10 Department of Computer Science, Vrije Universiteit Amsterdam, Amsterdam, Noord-Holland The Netherlands; 11 St. Pölten University of Applied Sciences, St. Pölten, Austria; 12 FIZ Karlsruhe – Leibniz Institute for, Karlsruhe, Germany; 13 Karlsruhe Institute of Technology, Karlsruhe, Germany; 14 UAS St. Pölten, St. Pölten, Niederösterreich Austria; 15 Vienna University of Economics and Business, Vienna, Wien Austria; 16 VU Amsterdam, Amsterdam, The Netherlands; 17 ADAPT Centre, Trinity College Dublin, Dublin, Ireland; 18 Dipartimento di Informatica, Università di Salerno, Fisciano, Italy; 19 ACT OR S.r.l., Rome, Italy

**Keywords:** Knowledge graph, Query builder, Data visualization, SPARQL & SQL queries, Faceted search, Natural language queries

## Abstract

While Open Data (OD) publishers are spur in providing data as Linked Open Data (LOD) to boost innovation and knowledge creation, the complexity of RDF querying languages, such as SPARQL, threatens their exploitation. We aim to help lay users (by focusing on experts in table manipulation, such as OD experts) in querying and exploiting LOD by taking advantage of our target users’ expertise in table manipulation and chart creation.

We propose QueDI (Query Data of Interest), a question-answering and visualization tool that implements a scaffold transitional approach to 1) query LOD without being aware of SPARQL and representing results by data tables; 2) once reached our target user comfort zone, users can manipulate and 3) visually represent data by exportable and dynamic visualizations. The main novelty of our approach is the split of the querying phase in SPARQL query building and data table manipulation.

In this article, we present the QueDI operating mechanism, its interface supported by a guided use-case over DBpedia, and the evaluation of its accuracy and usability level.

## Introduction

Open Data (OD) providers mainly opt for publishing data by non-proprietary formats (such as CSV) [8]. As a publisher, it requires minimum effort due to the easiness of the data format, and, as a consumer, it provides free access to resources [3, 4]. To fully benefit from OD, data should also provide their context to create new knowledge and enable data exploitation [3]. Therefore, data providers are strongly encouraged to move published datasets from 3-stars to 5-stars, i.e., to publish data in RDF format and interlink them to other resources to provide context [4]. 5-stars data are also referred to as Linked Open Data (LOD). Among the several different definitions of a Knowledge Graph (KG), we adopt the definition according to a KG is achieved by attaching to LOD their schema (i.e., an ontology) [15]. LOD facilitate innovation and knowledge creation from the publishing perspective [3, 4]. However, from the consumption point of view, LOD exploitation is threatened by the complexity of their querying languages. Even if SPARQL [22] has been recognized as the most common query language for RDF data, it proves to be too challenging, mainly for lay users [7, 9].

The *problem* we aim to solve is how to help potential users of the semantic web in *easily* accessing LOD (without requiring the explicit usage of SPARQL) and in exploiting the retrieved data. We aim to mainly focus on experts in data table manipulation and chart creation. It is not a strong limitation since many data visualization tools start from CSV files (or in general data tables). Thus, we can refer to our target users as experts in data table manipulation, and we aim to guide them in manipulating LOD through their tabular representation.

We propose a *transitional approach* where users are guided from LOD querying to our target user comfort zone, i.e., a tabular representation of data, table manipulation, and chart generation. As a result, we implement this transitional approach in *QueDI* (Query Data of Interest) that allows users to build queries step-by-step with an auto-complete mechanism and to exploit retrieved results by exportable and dynamic visualizations. Users can query LOD without explicitly creating SPARQL queries, and it is not required any previous knowledge of queried data. Users can inspect the nature of data by inspection, using natural language (NL) and query building. Query builders are about trading off *usability* of the proposed mechanism and its *expressivity*. We opt for a faceted search interface (FSI) enhanced by a NL query to extract results that reply to users’ requests and by modelling them as a table. By this approach, we cover Basic Graph Patterns (BGPs), such as path traversal, union, filters, negation, and optional patterns. The component that implements this approach (corresponding to the first step of our workflow) will be referred to as ELODIE (Extractor of Linked Open Data of IntErest) (pronounced elədē). When users are satisfied with the retrieved results, they can move to the second step of our workflow, i.e., the table manipulation, to perform aggregations, filtering, sorting; finally, they can represent knowledge by dynamic and exportable visualizations during the third and last step of our scaffolded approach. Therefore, by combining the expressivity of ELODIE and table manipulation, we cover SELECT queries which results can always be represented as a table, BGPs directly in SPARQL, sorting, GROUP BY, aggregation operators and filtering by table manipulation.

The Research Questions (RQs) we aim to reply are:

RQ1.: *Does the proposed approach lose in accuracy?* We aim to compare our two-phase approach (SPARQL queries building and table manipulation) with the formulation of SPARQL queries only exploiting SPARQL query building.
RQ2.: *Do lay users (users without technical skills in the Semantic Web technologies) consider usable the ELODIE operating mechanism and its interface?*
RQ3.: *Are lay users able to quickly learn how to exploit ELODIE in retrieving data of interest?*

The main contributions of this article are:

- the proposal of a transitional approach to guide table manipulation experts in exploiting LOD by relying on their abilities in data manipulation and chart creation;
- the implementation of the proposed approach in QueDI, a guided workflow composed of 1) ELODIE, a SPARQL query builder provided on a FSI enhanced by a NL query to query LOD without explicitly using SPARQL; 2) data table manipulation and 3) chart creation.

The main *novelties* of our proposal are 1) the provision of a *querying mechanism articulated in two steps*: a SPARQL query building phase to retrieve results from LOD followed by a SQL building phase to manipulate retrieved results; 2) a *guided workflow* from data querying to knowledge representation instead of the juxtaposition of visualization mechanisms to query builders.

The rest of this article is structured as follows: in Sect. 2, we overview related work on making semantic search more usable, and we mainly focus on the trade-off between usability and expressivity they propose; in Sect. 3, we present challenges in querying LOD, the QueDI implementation overview, and a navigation scenario on DBpedia; in Sect. 4, we estimate the QueDI accuracy and expressivity by a standard benchmark dataset (QALD-9 on DBpedia) and its usability (also including temporal aspects); finally, we will conclude with some final remarks and future directions.

## Related Work

During the past years, several different approaches have been proposed to hide the complexity of SPARQL and enable query building. Users can query KG by creating graph-like queries (such as FedViz [23], RDF Explorer [21]) or visual query formulation (e.g., OptiqueVQS [19]), they can interact with facets (e.g., SemFacet [2]), also enhanced by keyword search interfaces (such as SPARKLIS [10] and Tabulator [5]), they can be helped by query completion (such as YASGUI [16]), users can work with summarization approaches (such as Sgvizler [18]), or a combination of them. The expressivity level of the querying method can be affected by the interaction model, the required usability, the efficiency. Some tools support users not only in retrieving data but also in visualizing them. We will focus on tools that combine data querying and visualization.

In Table 1, we provide an overview of the considered tools by presenting a schematic comparison of query building mode, expressivity, and the need for SPARQL awareness by users. Moreover, we also consider the provided visualization mode, and if customization and export are enabled.

Tabulator [5] leads to query (and modify) KGs without SPARQL awareness. Users can interact with an FSI where predicate/object pairs are reported for each focused element, and the user can recursively follow paths by choosing element by element. Besides the tabular representation of retrieved results, Tabulator provides basic visualizations: if results contain temporal or geographical information, the user can create timelines and/or maps. It is not mentioned if the realized visualization can be customized and/or exported.

**Table 1.** Comparison of interfaces to query KGs and visualize the retrieved results. For each work we report 1) the year of publication, 2) the interaction mode, expressiveness and the awareness of SPARQL for the Query builder, 3) visualization mode and the possibility to customize and export the visualization. $\sim$ means that the feature is partially covered; empty cells mean that the feature is not clarified by the author(s).

Tool	Year	Query builder			Visualization		
		Mode	Expressivity	SPARQL awareness	Mode	Custom	Export
TABULATOR [5]	2006	facet	Path Traversal	$\times$	time, map		
NITELIGHT [17]	2008	graph	SPARQL 1.0−	$\sim$	time, map		
VISINAV [12]	2010	facet$+$ keywords	BGPs	$\times$	time, map		$\checkmark$
Sgvizler [18]	2012	text	SPARQL	$\checkmark$	Google Charts	$\checkmark$	
VISU [1]	2013	text	SPARQL	$\checkmark$	Google Charts		
Visualbox [11]	2013	text	SPARQL	$\checkmark$	chart, map, time	$\checkmark$	$\checkmark$
Rdf:SynopsViz [6]	2014	form	BGPs−	$\times$	chart, treemap, time	$\times$	$\checkmark$
YASGUI	2017	text	SPARQL 1.1	$\checkmark$	Google Charts	$\checkmark$	$\checkmark$
SPARKLIS [10]	2018	facet$+$NL	SPARQL−	$\times$	Google Charts $+$ map, image	$\checkmark$	$\checkmark$
WQS [14]	2018	form	BGPs	$\times$	chart, map, time, image, graph	$\checkmark$	$\checkmark$
**QueDI**	2020	facet$+$NL	BGPs$+$	$\times$	chart, time, image, map	$\checkmark$	$\checkmark$

NITELIGHT [17] is a tool to create graphical SPARQL queries. Authors declare that it is intended for users that already have a SPARQL background since the complexity and the structures of SPARQL patterns are not masked during the query definition. A keyword browser supports the query formulation to lookup classes and properties of interest. The output of the query can be visualized as a map and/or timeline. It seems that the resulting visualization can neither be customized and exported.

VISINAV [12] leads users in looking up for a keyword of interest, without knowing the underlying data modelling. The keyword is literally searched into the KG, without extending it with synonyms and related terms. Starting from retrieved results, the user can follow paths and select facets to manipulate and extend the result set. Furthermore, VISINAV supports basic temporal and spatial visualizations. While the export seems to be provided, it is not clarified if the customization can be performed.

VISU [1] and Sgvizler [18] are both query builders and data visualization tools. Users can interact with a single or multiple SPARQL endpoints by directly using SPARQL (therefore users are SPARQL aware), manipulate the resulting table, and create customizable and exportable visualization by Google Charts. While Sgvizler is general-purpose, VISU is bound for university data.

Visualbox [11] is an environment to query KGs by SPARQL and view results by a set of visualization templates (called filters). These filters can be downloaded and wrapped in other hyper-textual documents, such as blogs or wikis.

rdf:SynopsViz [6] provides faceted browsing and filtering over classes and properties inferring statistics and hierarchies from data without requiring any further interaction by the user. Once data have been retrieved, users can visualize them by charts, treemaps, timelines according to data and needs. Visualization can be exported, but not customized by the user.

YASGUI [16] guides users in querying KGs by directly using SPARQL and visualize data through Google Charts. The query builder is enhanced by auto-completion, while the integration with Google Charts provides customizable and exportable visualizations.

SPARKLIS [10] is a query builder based on a faceted search and a natural language interface. Within the tool, it offers basic visualizations, such as maps and image viewers. Furthermore, it is integrated with YASGUI and, thus, it inherits its visualization approach. Unlike YASGUI, it can mask the complexity of SPARQL, without losing its expressiveness.

Wikidata Query Service (WQS) [14] is bound for Wikidata; it leads to the creation of queries by a form-based interface, and it provides several different visualization modes, such as charts, maps, timelines, image viewers, and graphs.

Our proposal, QueDI, is a guided workflow from KG querying to data visualization. Users can query LOD by FSI enhanced by an NL query. The interface masks an automatic and on-the-fly generation of SPARQL queries. By only considering the SPARQL query generation phase, we cover BGPs. By also considering the dataset manipulation phase, we cover aggregation and sorting. This consideration justifies that the expressivity of QueDI is more than BGP. Finally, customizable and exportable visualization can be created. Users can export the visualization as an image or as a dynamic and live component that can be embedded in any hyper-textual page, such as HTML pages, WordPress blogs and/or Wikis.

The main *difference* with the previous works is the split of the expressivity of the query building phase in an implicit creation of SPARQL queries over KGs and by direct manipulation of datasets to perform aggregation and sorting.

## QueDI: A Guided Approach to Query and Exploit LOD

### Linked Open Data Querying Challenges

The main challenges posed by querying LOD are:

- **technical complexity of SPARQL**: SPARQL is extremely expressive but writing SPARQL queries is an error-prone task, and it is largely inaccessible for lay users;
- **hard conceptualisation**: data can be modelled by domain-specific schema, or they can be domain-agnostic. Therefore, it may not be easy to conceptualize the data that users are querying;
- **heterogeneity** in data modelling: this issue is strongly related to the difficulties in conceptualization. Since different endpoints can use different vocabularies and ontologies, it is hard to figure out the terminology to use in posing questions;
- **scalability** to manage (potential) huge amount of data;
- **portability** to different endpoints;
- **readability** of queries and retrieved results;
- **intuitive use** in deriving results by few and clear clicks.

By overviewing the QueDI features and its operating mechanism, we will point our solution to these challenges.

### QueDI Overview

**Fig. 1.**
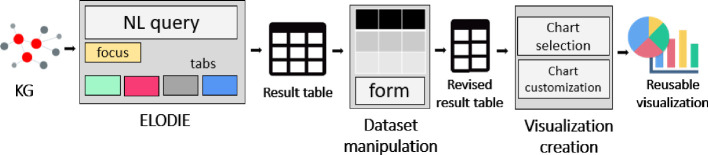
It represents the QueDI guided workflow into three phases: the *SPARQL query building* implemented by ELODIE to query KGs and organize results by a tabular format; the *dataset manipulation* to refine the table and the *visualization creation* where the acquired knowledge is graphically represented.

In this section, we present the QueDI system, whose goal is to enable lay users with a background in data table manipulation to query KGs and visualize the retrieved results. To guide users in the entire workflow, we split the querying and exploitation process into three steps (Fig. 1). Each step has a clear objective, and we aim to guarantee few and clear interactions a time to provide an *intuitive use.* The implemented steps of our scaffold approach are:

- **Dataset creation** the user starts from a SPARQL endpoint and can query the KG. This step aims to create the dataset of interest, i.e., a dataset that replies to the question of interest, without requiring any expertise in SPARQL. ELODIE implements this phase. By representing the SPARQL query results as a data table, we move from LOD to the conform zone of the experts in data manipulation. It represents the transitional approach from LOD to data table representation.
- **Dataset manipulation** when the user is satisfied with the retrieved data, he/she can start the manipulation of the dataset to refine its information and to make it compliant with the desired visualization. In this stage, we exploit the skills of our target in data table manipulation: users can refine results, aggregate values, and sort columns. The goal of this step is to clean the data table and make it compliant with the visualization requirement.
- **Visualization creation** (exportable and reusable) visualizations can be created and customized. This step realizes the immediate gratification for information consumers of seeing the results of their effort in a concrete artefact. We provide the customization based on the user’s preferences and the export to enable the reuse also out of QueDI.

The entire workflow implemented in QueDI takes place on client-side, without any server-side computation. QueDI is released open-source on GitHub^1^. To see how QueDI works, you can access to the online demo^2^. Quick tutorials^3^ are available on YouTube.

***ELODIE - Dataset Creation Phase.*** ELODIE is a SPARQL query builder provided by an FSI and enhanced by an NL query. First, the user has to select the endpoint of interest among the provided suggestions. The supported endpoints, at the moment, are DBpedia^4^, also the Live version^5^, the French endpoint *Persée*^6^, the Italian endpoint *Beni Culturali*^7^, and the Chilean endpoint *National library of Chile*^8^. By default, ELODIE will query DBpedia. Then, we can move to the querying phase.

**Fig. 2.**
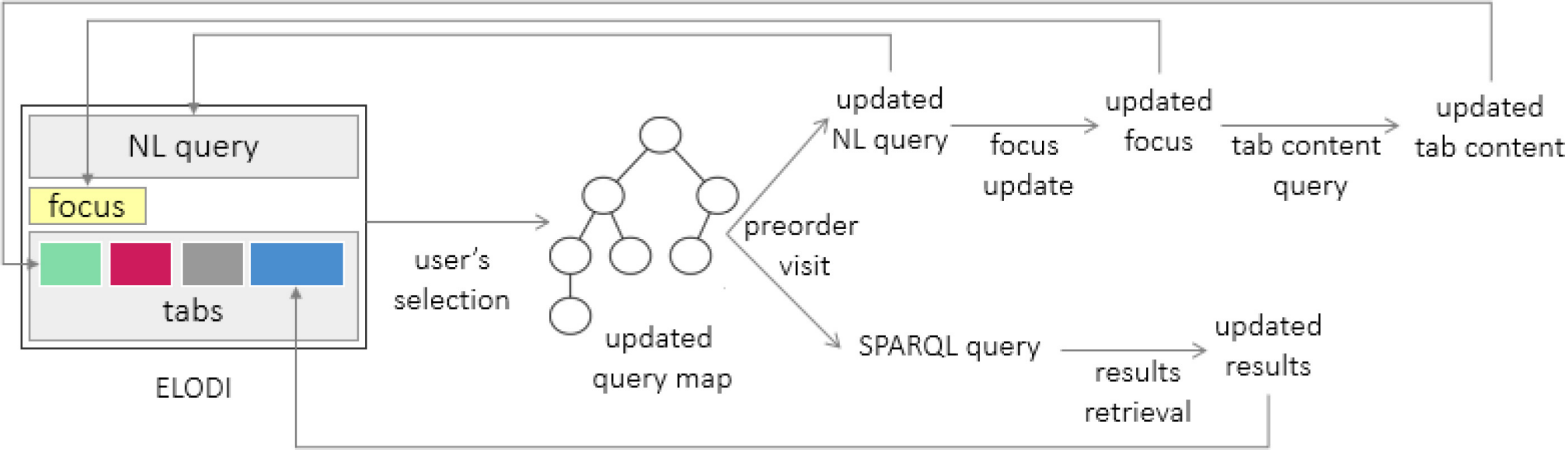
*Operating mechanism of ELODIE.* Starting from a user’s selection, first, the map that models the user query is updated, and then, by a pre-order visit of this tree, both the NL and the SPARQL queries are generated. When the NL query is updated, also the related box in the ELODIE interface will be updated. The focus will reflect the last added element. According to the focus, ELODIE updates the tab content by querying the SPARQL endpoint. About the SPARQL user query, when the results are retrieved, the results table is updated.

Figure 2 represents the operating mechanism of ELODIE: the *user query* and the *focus* determine the state of the system. The NL query represents the user query, therefore herein NL query and user query will be used as synonyms. While the *user query* represents the query under construction, the *focus* represents the insertion position for applying query transformation. According to the focus, *concepts* (i.e., classes), *predicates* (i.e., relations) and resources are retrieved from the endpoint and organised in *facets* (also referred to as *tabs*). More in detail, all the sub-classes that can refine the focus are listed in the classes tab; all the predicates that have the focus as subject (direct predicates) or as the object (reverse predicates) are listed in the predicate tab.

Users can go on in the query formulation by selecting any element listed in the tabs. Therefore, the user query is iteratively defined, and the content of the queried source is discovered by inspection. It solves the problem of *conceptualised data* since users have access to valid options (referred to as *suggestions*) without explicitly asking for them. Suggestions are retrieved by path traversal queries, generic enough to be used to retrieve data from any endpoints, by solving the *portability* issue. At each query refinement, the map that models user interactions is updated by modifying the focus neighbourhood. Then, by a pre-order visit of the map, both the NL and the SPARQL queries are generated. While the NL query will be used to verbalize the user’s interactions, the SPARQL query will be posed against the SPARQL endpoint to retrieve the user query’s results. Once retrieved, results are organized by a tabular view. The last selected element behaves as the new focus, and, according to it, all the facets are consistently updated by querying the endpoint. This process is repeated to each user selection.

Thanks to the FSI, users are guided *step-by-step* in the query formulation. At each step, ELODIE provides a set of suggestions (concepts, predicates, operator, results) to go on in the query formulation by preventing empty results. A clarification is needed: empty results are a real desired results in a complete KG interpreted as a close world. Since common KGs are usually incomplete, empty results can be interpreted either as a real desired result or as missing information. As we can not automatically distinguish them, we prevent empty results by providing all the navigable edges outgoing from the focus as a suggestion. In other words, suggestions are focus-dependent. This exploratory search provides an *intuitive guide* in query formulation. Once a suggestion has been selected, it will be incorporated and verbalized into the current user query. It makes ELODIE a Query Builder, without asking for the SPARQL knowledge. SPARQL is completely masked to the final users by providing a solution to the *technical complexities of SPARQL*.

The query, suggestions, and results are verbalized in NL to solve the *readability* issue. Therefore, instead of showing URIs, we retrieve resources label. Class and predicate labels are obtained by looking for rdfs:label predicate attached to the retrieved results and by asking for the label in the user language. If these labels are missing, ELODIE looks for the English label. If also this attempt fails and resources are not attached to rdfs:label, the URL local names are exploited as labels. Suggestion labels are contextualized by phrases. For instance, instead of showing author as a predicate, the predicate label is wrapped into a meaningful phrase, such as that has an author. The user query always represents a complete and meaningful phrase. Therefore, ELODIE is a kind of NL interface. However, it is worth to notice that users cannot freely input the query, but ELODIE is provided with a *controlled* NL query used to verbalize the iteratively created user query. It makes query formulation less spontaneous and slower instead of directly writing the query in NL, but it provides intermediate results and suggestions at each step, prevents empty results, and avoids ambiguities issues of free-input NL query and out-of-scope questions. Queries and suggestions can be verbalized in English, Italian, and French, and new supported languages with the same syntax (such as Spanish) can be easily incorporated.

Only a limited number of results and suggestions are retrieved to address *scalability* issues. However, this limit can be freely changed by users. The main drawback of limited suggestions is that it can prevent the formulation of some queries. Therefore, we propose an intelligent auto-completion mechanism at the top of each suggestion list. At each user keystroke, it filters the corresponding suggestion list for immediate feedback. If the lists get empty, the list of suggestions is re-computed by asking suggestions that include the user filter.

To promote *portability*, ELODIE is entirely based on Web standards: the entire application is written in Javascript and the interface with HTML/CSS, with *zero configuration*. It only requires Cross-Origin Resource Sharing (CORS) enable SPARQL endpoint URL, i.e., a specification that enables truly open access across domain-boundaries.

As already stressed, ELODIE enables the formulation of SELECT queries (that enables the provision of results in a tabular format) by covering BGPs.

***Dataset Manipulation.*** This phase implements a SQL query builder provided by a form-based interface. Users can *select* columns of interest, perform *aggregation*, *filtering*, *sorting*. Data manipulation is enabled by a form-based interface where users can choose the column to affect, the operation of interest (such as group by or filters), and complete it by the required parameter(s). For instance, he/she can ask for removing empty cells from a column, remove all values but numbers, filter a column by number or string operations, group the table by column values, aggregate values by counting or summing them, computing the average or detecting the minimum or maximum value. The sorting is intuitively enabled on the top of each column. These patterns enhance the BGPs of ELODIE. By aggregation we mean that users can perform group by and compute statistics of retrieved data, such as count, average, sum. By filtering, we mean that users can remove empty cells or remove cells according to textual and numeric filters, such as contains for strings and less than for numbers. By each user interaction, a SQL query is automatically created to update the result table. In this step also, the query formulation is completely masked to the user.

***Visualization Creation.*** This step implements the exploitation phase, where users are guided in representing the acquired knowledge by charts. Besides proposing the realization of mere images, we realized a mechanism to produce dynamic artefacts that can be embedded in any blog, web page as an HTML5 component. Instead of wrapping the dataset in the chart, we embed the query to retrieve and refine the dataset in the representation. It always ensures up to date results. Therefore, if data in the queried endpoint change, also their visual representation will change as well. According to the *guidance* principle, users are provided with a vast pool of charts, such as timelines, maps, media-players, histograms, pie charts, bar charts, word clouds, treemaps. Only charts compliant with the provided data will be enabled. According to the chosen visualization mode, users can customize both the chart content and its layout. Then, the realized chart can be download as an image or as a dynamic component.

### Navigation Scenario on DBpedia

We detail a navigation scenario using QueDI on DBpedia. Table 2 contains iterative queries as verbalised by QueDI of a navigation scenario that retrieves the *geographical distribution of the Italian architectural structures*. At each step, the bold part represents the last suggestion selected by the user and the underlined part represents the query focus. Suggestions can be classes (e.g., city), direct and inverse properties (respectively, has a thumbnail and is the location), operators (e.g., that is equals to) and resources (e.g., dbr:Italy^9^).

**Fig. 3.**
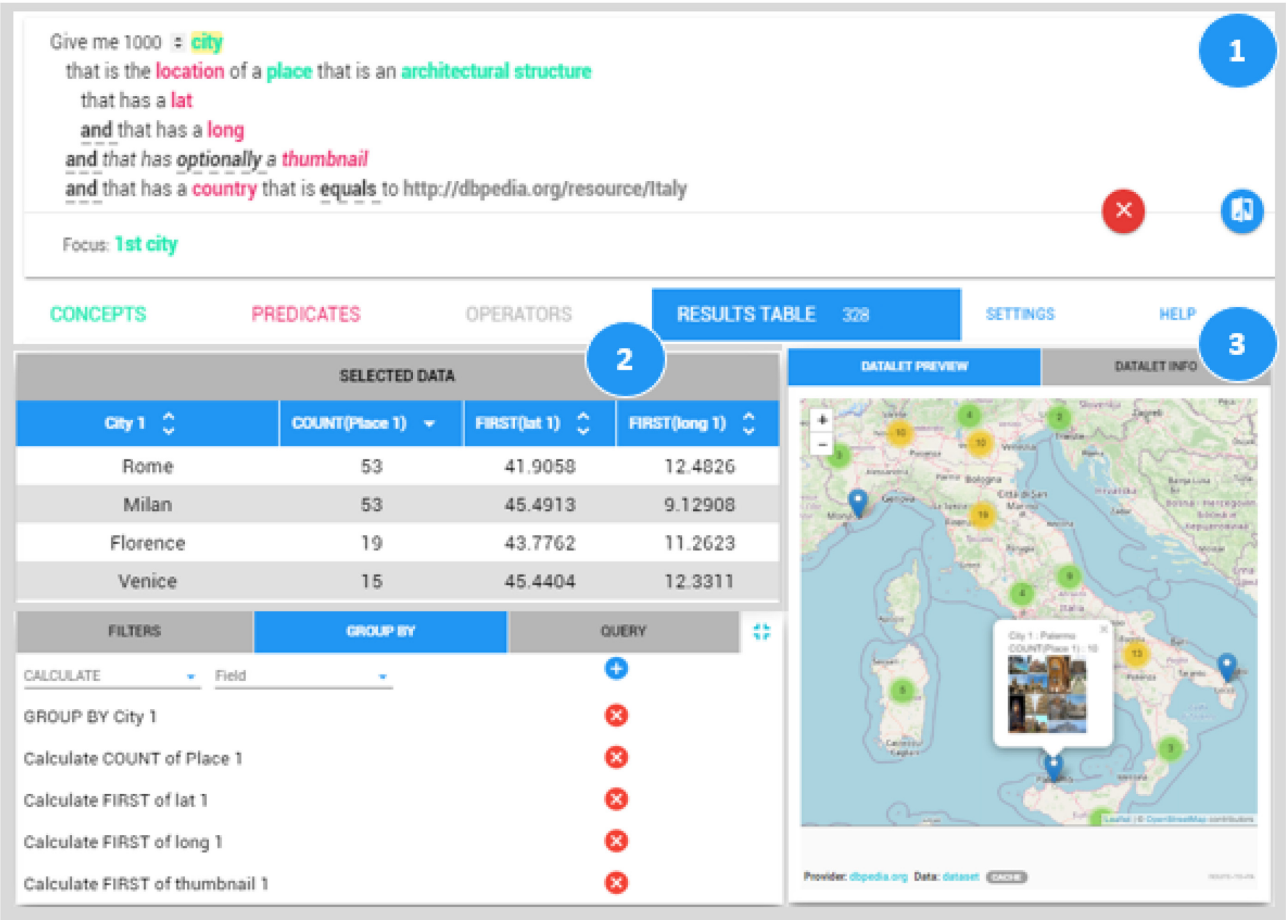
The 1st component represents the user query of our navigation scenario as formalized by ELODIE to retrieve all the Italian architectural structures and related geographic information; the 2nd component represents the aggregated version of the results table to obtain the geographical distribution of the architectural structures; while the 3rd component reports the visual representations of the geographical distribution of structures on the Italian map. (Color figure online)

Fig. 3 is a collage of screenshots of the different steps of the QueDI workflow. On the top (Fig. 3.1), there is the user query at the end of its formulation by ELODIE. The focus is highlighted in yellow in the user query, and it is verbalized below the user query. When the user is satisfied with the retrieved results, he/she can move to the second step, i.e., the dataset manipulation (Fig. 3.2). In this step, we group data by city and count the architectural structures in each group. In other words, we perform data aggregation. We also sort data by the number of structures. Now, we are ready to visualize the retrieved results and represent the achieved knowledge by an exportable visual representation. The third part (Fig. 3.3) represents the geolocalized distribution of architecture structures on the Italian map.

## Evaluation

### Accuracy, Expressivity and Scalability over QALD-9

In this section, we evaluate the accuracy, expressivity, and scalability of QueDI. As stated before, we split the query formulation into two phases, i.e., a SPARQL query generation to retrieve results of interest and a SQL query generation to aggregate and sort results. Thus, we want to verify if (and in which cases) the accuracy is compromised. We hypothesize that the accuracy is affected only when the complete set of query results is so huge that the queried endpoint does not return all the results or our platform can not manage them. We want to assess the expressivity level by testing QueDI on standard benchmark for question answering and its scalability when tested against real KGs, such as DBpedia.

**Table 2.** *A navigation scenario in ELODIE over DBpedia.* Underlined words represent the focus, while phrases in bold represent the last selected suggestion.

**Step**	**Query**
1	Give me something
2	Give me a **city**
3	Give me a city **that is the location of** **something**
4	Give me a city that is the location of a **place**
5	Give me a city that is the location of a place **that is an** **architectural structure**
6	Give me a city that is the location of a place that is an architectural structure **that has a** **lat**
7	Give me a city that is the location of a place that is an architectural structure that has a lat and **that has a** **long**
8	Give me a city that is the location of a place that is an architectural structure that has a lat and that has a long
9	Give me a city that is the location of a place that is an architectural structure that has a lat and that has a long and **that has a** **thumbnail**
10	Give me a city that is the location of a place that is an architectural structure that has a lat and that has a long and that has **optionally** a thumbnail
11	Give me a city that is the location of a place that is an architectural structure that has a lat and that has a long and that has optionally a thumbnail
12	Give me a city that is the location of a place that is an architectural structure that has a lat and that has a long and that has optionally a thumbnail and **that has a** **country**
13	Give me a city that is the location of a place that is an architectural structure that has a lat and that has a long and that has optionally a thumbnail and that has a country **that is equals to** http://dbpedia.org/resource/Italy

**Dataset**. We tested QueDI, mainly focusing on ELODIE and the data manipulation phase, on the QALD-9 challenge dataset^10^. This dataset behaves as benchmarks in comparing NL Interfaces. We took into account the QALD-9 DBpedia multilingual test set^11^. For each of the 150 testing questions over DBpedia, it contains the English (among the multi-language options) verbalization of each question, the related SPARQL query, and the collection of results.

**Experiment**. We evaluated the minimum number of interactions and the related needed time starting from the empty query (i.e., Give me something). Since we aim to assess the accuracy of our two-step querying approach, the expressivity of QueDI, and the scalability on real datasets and *not* the usability, we aim to minimize the exploration and thinking time required by users to conceptualize queries. Thus, we both consider the English NL formulation of the query and the related SPARQL query while performing them on QueDI. The measured time represents the best interaction time for a trained and focused user in performing questions on QueDI. In real use, interaction time will increase according to unfamiliarity with QueDI and the queried dataset and lack of focus in exploratory search. We will consider usability and interaction time in Sect. 4.2.

**Results**. We estimate accuracy, precision and F-measure, both for each question (*macro-measure*) and for the entire dataset (*micro-measure*). In Table 3, we report achieved results. The actual code used for the comparison and the results are provided on GitHub^12^. The challenge report [20] contains also results achieved by participants, that can be used for tool comparison.

**Table 3.** It reports the micro and macro precision, recall and F1 score obtained by testing QueDI on QALD-9 testing dataset.

	Precision	Recall	F1		Precision	Recall	F1
Macro results	0.75	0.95	0.83	Micro results	0.92	0.93	0.92

***Expressivity.*** With QueDI, we can answer 143/150 questions. Not supported patterns cause the failures, i.e., make computation by SPARQL operator (3/7 cases), field correlation and not exist), and, in 2/7 cases, too many results.

***Accuracy.*** In 20/143 cases, we both exploited ELODIE expressivity and data manipulation features. By considering the queries that requires further refinement, sorting or aggregates, we observe that: in 8/20 cases we perform a group by to remove duplicates; in 4/20 cases we perform group by, count as aggregation and sort; in 8/20 cases only sorting is required. It is worth to notice that ELODIE returns the count of table tuples without requiring any further interaction. Only one failure is caused by a too wide pool of results (*all books and their numbers of pages*) that QueDI is not able to manage. In conclusion, we can consider that by splitting the querying phase into two steps, we only lose accuracy when the desired query is too wide and/or the desired results are too much to be first collected and then refined (RQ1).

***Scalability.*** By considering the interaction time for the 143 successful questions, we observe that: more than half of the questions (75/143) can be answered in less than 40 s (with 30 s as median and average time); 115 of them can be replied in less than 60 s (with 0,4 as average time and 0,37 as median time); only 6 of them requires a time that lies between 2 min and 3 min and a half (median time 40 s and average time 60 s).

### Usability

We estimate the usability and the execution time in real use by providing a list of tasks to inexperienced participants and by collecting results of a standard questionnaire (we used SUS [13]) to assess the system *usability* (to reply to RQ2) and by comparing the needed *time* of lay users with the execution time of focused expert in accomplishing the same tasks (to reply to RQ3). Besides SUS, we also ask participants to provide *subjective* perception of the complexity and usability of QueDI, by estimating the perceived complexity in replying questions by QueDI, if the few indications provided by the training phase enable them to effectively using ELODIE, and if the effort and needed time to interact with QueDI is reasonable.

**Sampling.** The users involved in the testing phase are 23 in total: 11 with skills in computer science and dataset manipulation (we involved both students still studying and already graduated) and 12 lay users, without any technical skill in querying language and heterogeneous background.

**Experiment.** We structured the evaluation as follows:

- we performed *15 min* of *training* to provide users with the opportunity to become familiar with QueDI (in particular with ELODIE) and the queried data by performing guided examples and by answering to queries of incremental complexity. All users were not aware of QueDI in advance;
- *testing phase*: six tasks (Table 4) are submitted in the Italian language asking for the use of DBpedia. The tasks are of incremental complexity, as for the training phase. For each task, the user reported the completion time and filled in an After Scenario Questionnaire (ASQ) using a Yes/No answers to evaluate 1) the degree of the perceived difficulty of the task by performing it through ELODIE, 2) if the time to complete the task is reasonable, 3) if the provided knowledge in the training phase is sufficient to complete the task.
- in conclusion, we asked for the fulfilment of a *final questionnaire* to evaluate i) the user satisfaction based on a Standard Usability Survey (SUS [13]) and ii) the interest in using and proposing the tool by a Behavioural Intentions (BI) survey. The questions of the BI survey are: i) “*I will use the system regularly in the future*”; ii) “*I will strongly recommend others to use the system*” and users can use a 7-point scale to reply. In the end, the participants in the evaluation study were free to suggest improvements, report the main difficulties, and the strengths of QueDI as open questions.

**Table 4.** Tasks provided during the evaluation of the usability of QueDI.

Task #	Query
Task 1	The Italian museums
Task 2	The games with at least 2 players
Task 3	The presenters who are the presenter of a TV Show
Task 4	The female scientists born and dead in Germany
Task 5	The athletes which are not dead
Task 6	The artists born in the same place of an athlete

**Usability.** The SUS score is 70 for the first group and 68 for the second one. According to the SUS score interpretation, all the values at least equal to 68 classify the system as *above the average*. That means that QueDI is considered *usable* both for technical and lay users (RQ2). In the open questions, it is clear that the perceived usability is closely related to the training phase: users - especially not experienced ones - need initial training to get familiar with KGs and their modelling. About BI, both the groups reached an average score of 5 in both the questions, i.e., there is an overall intention to reuse and propose ELODIE.

**Fig. 4.**
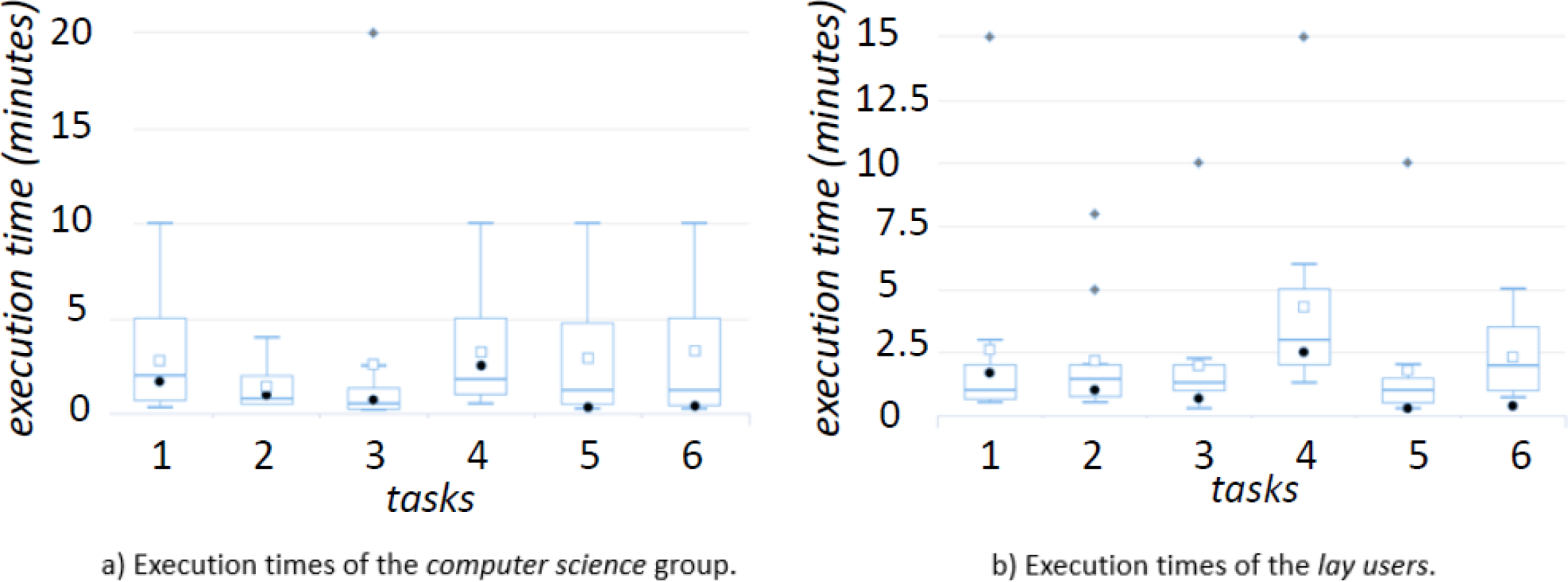
The time is reported on the y-axis, the tasks on the x-axis. The square icons represent the average score. The black dots represent the *optimal* value. The grey diamonds represent outliers.

**Execution time.** For each group, we consider the execution time compared to the time needed to one expert of the field (also familiar with QueDI) - hence called *optimal* value. The results related to the first group - the Computer Science experts - are reported in Fig. 4a. While the results related to the second group - the lay users - are reported in Fig. 4b.

In all the queries - but the last query for the second group - the minimum time needed by the participants either matches the *optimal* one or it is even better. It is a surprising result, and it means that there are users (at least one in each group) able to get familiar with QueDI and learning how to use it in a short time (RQ3). About the outliers, in the open questions, it is evident that the main difficulties are in “*finding the exact way to refer to an asked predicate or concept*” (reported by 6 out of 23 users). The participants suggest to “*insert inline help, tool-tips to help the users during the usage, examples of usage*” (reported by 9 out of 23 users). The start is considered a small obstacle to face: “*After a bit of experience, the system is pretty easy to use*” (reported by 6 out of 23 users).

## Conclusion and Future Work

In this article, we present a transitional approach to bring closer the Semantic Web technologies and the community of tabular data manipulation and representation by enabling querying and visualization of LOD. We implement the proposed approach in QueDI, a guided workflow from data querying to their visualization by dynamic and exportable data representations. We propose to split the querying phase in SPARQL queries building and data table manipulation and we loose in accuracy only when results are too much to be first retrieved and then filtered (RQ1). The 70 score according to the SUS questionnaire reports that QueDI is considered usable by lay users (with and without table manipulation skills) (RQ2). The needed time by users with computer science background to interact with ELODIE is almost indistinguishable by the execution time of focused users, experts in QueDI features (RQ3).

***Future Work.*** The described evaluation is a preliminary experiment to assess QueDI performance. We are defining a comparison between QueDI and state of the art. We aim to enrich the proposed endpoints by also considering the integration of a proxy to overcome the issue of not CORS-enabled endpoints. Moreover, we aim to further simplify the exploratory search in retrieving suggestions by also considering synonyms and alternative forms of the queried keywords.

## Footnotes

1 QueDI on GitHub: https://github.com/routetopa/deep2-components/tree/master/controllets/splod-controllet.

2 Online demo of QueDI: https://deep.routetopa.eu/deep2/COMPONENTS/controllets/splod-visualization-controllet/demo.html.

3 YouTube tutorials of QueDI: https://youtu.be/e_o32GP-l1c.

4 https://dbpedia.org/sparql.

5 http://live.dbpedia.org/sparql.

6 http://data.persee.fr/sparql.

7 http://dati.culturaitalia.it/sparql.

8 https://datos.bcn.cl/sparql.

9 dbr is the prefix corresponding to http://dbpedia.org/resource/.

10 QALD-9 challenge https://project-hobbit.eu/challenges/qald-9-challenge/.

11 QALD-9 dataset https://github.com/ag-sc/QALD/blob/master/9/data/qald-9-test-multilingual.json.

12 https://github.com/mariaangelapellegrino/QueDI_evaluation.

## References

[1] Alonen M, Kauppinen T, Suominen O, Hyvönen E, Cimiano P, Fernández M, Lopez V, Schlobach S, Völker J (2013). Exploring the linked university data with visualization tools. The Semantic Web: ESWC 2013 Satellite Events.

[2] Arenas, M., Cuenca Grau, B., Kharlamov, E., Marciuska, S., Zheleznyakov, D., Jimenez-Ruiz, E.: SemFacet: semantic faceted search over yago. In: Proceedings of the 23rd International Conference on World Wide Web, pp. 123–126 (2014)

[3] Bauer, F., Kaltenböck, M.: Linked Open Data: The Essentials - A Quick Start Guide for Decision Makers, vol. 710 (2011)

[4] Berners-Lee, T.: 5 star open data (2012). https://5stardata.info/en/. Accessed April 2020

[5] Berners-Lee, T., Hollenbach, J., Lu, K., Presbrey, J., Prud’hommeaux, E., Schraefel, M.M.C.: Tabulator redux: browsing and writing linked data. In: Proceedings of the WWW Workshop on Linked Data on the Web, LDOW (2008)

[6] Bikakis N, Skourla M, Papastefanatos G, Presutti V, Blomqvist E, Troncy R, Sack H, Papadakis I, Tordai A (2014). rdf:SynopsViz – a framework for hierarchical linked data visual exploration and analysis. The Semantic Web: ESWC 2014 Satellite Events.

[7] Damljanovic D, Agatonovic M, Cunningham H, Aroyo L, Antoniou G, Hyvönen E, ten Teije A, Stuckenschmidt H, Cabral L, Tudorache T (2010). Natural language interfaces to ontologies: combining syntactic analysis and ontology-based lookup through the user interaction. The Semantic Web: Research and Applications.

[8] European Data Portal: Metadata Quality Assurance (2020). https://www.europeandataportal.eu/mqa/?locale=en. Accessed April 2020

[9] Ferré S (2014). SQUALL: the expressiveness of SPARQL 1.1 made available as a controlled natural language. Data Knowl. Eng..

[10] Ferré S (2017). SPARKLIS: an expressive query builder for SPARQL endpoints with guidance in natural language. Semant. Web.

[11] Graves, A.: Creation of visualizations based on linked data. In: Proceedings of the 3rd International Conference on Web Intelligence, Mining and Semantics, pp. 41:1–41:12 (2013)

[12] Harth A (2010). VISINAV: a system for visual search and navigation on web data. Semant. Web.

[13] Lewis JR, Sauro J, Kurosu M (2009). The factor structure of the system usability scale. Human Centered Design.

[14] Malyshev, S., Krötzsch, M., González, L., Gonsior, J., Bielefeldt, A.: Getting the most out of Wikidata: semantic technology usage in Wikipedia’s knowledge graph. In: Vrandečić, D., et al. (eds.) ISWC 2018. LNCS, vol. 11137, pp. 376–394. Springer, Cham (2018). 10.1007/978-3-030-00668-6_23

[15] Paulheim H (2016). Knowledge graph refinement: a survey of approaches and evaluation methods. Semant. Web.

[16] Rietveld L, Hoekstra R, Cimiano P, Fernández M, Lopez V, Schlobach S, Völker J (2013). YASGUI: not just another SPARQL client. The Semantic Web: ESWC 2013 Satellite Events.

[17] Russell, A.: NITELIGHT: a graphical editor for SPARQL queries. In: Proceedings of the International Conference on Posters and Demonstrations, vol. 401, pp. 110–111 (2008)

[18] Skjæveland MG, Simperl E (2015). Sgvizler: a javascript wrapper for easy visualization of SPARQL result sets. The Semantic Web: ESWC 2012 Satellite Events.

[19] Soylu A (2018). OptiqueVQS: a visual query system over ontologies for industry. Semant. Web.

[20] Usbeck, R., Gusmita, R.H., Ngomo, A.N., Saleem, M.: 9th challenge on question answering over linked data (QALD-9). In: 17th ISWC, pp. 58–64 (2018)

[21] Vargas, H., Aranda, C.B., Hogan, A.: RDF explorer: a visual query builder for semantic web knowledge graphs. In: Proceedings of the ISWC, pp. 229–232 (2019)

[22] W3C - World Wide Web Consortium: SPARQL query language for RDF (2008). https://www.w3.org/TR/rdf-sparql-query/. Accessed April 2020

[23] e Zainab, S.S., Saleem, M., Mehmood, Q., Zehra, D., Decker, S., Hasnain, A.: FedViz: a visual interface for SPARQL queries formulation and execution. In: International WS on Visualizations and User Interfaces for Ontologies and Linked Data (2015)

